# Antibiotic Adjuvant Potential of Selected Essential Oil Components Against Respiratory Pathogens: From Planktonic Synergy to Early-Stage Biofilm Inhibition

**DOI:** 10.3390/antibiotics15040403

**Published:** 2026-04-16

**Authors:** Viktória Lilla Balázs, Rita Filep, Edit Ormai, Lilla Radványi, Béla Kocsis, Erika Kerekes, Marianna Kocsis

**Affiliations:** 1 Department of Pharmacognosy, Faculty of Pharmacy, University of Pécs, 7624 Pécs, Hungary; filep.rita@pte.hu (R.F.); ormai.edit@gytk.pte.hu (E.O.); lilla.radvanyi@gytk.pte.hu (L.R.); 2 Department of Medical Microbiology, Medical School, University of Pécs, 7624 Pécs, Hungary; kocsis.bela@pte.hu; 3 Department of Biotechnology and Microbiology, Faculty of Science and Informatics, University of Szeged, 6726 Szeged, Hungary; kerekeserika88@gmail.com; 4 Department of Agricultural Biology, Institute of Biology, University of Pécs, 7624 Pécs, Hungary; kocsis.marianna@pte.hu

**Keywords:** antibiotic adjuvants, essential oil components, respiratory pathogens, biofilm

## Abstract

**Background:** Respiratory tract infections remain among the most common indications for antibiotic therapy and represent a major driver of antimicrobial resistance. The ability of respiratory pathogens to form biofilms further contributes to treatment failure and recurrence. This study aimed to evaluate the antibiotic adjuvant potential of selected essential oil components against clinically relevant respiratory bacteria and to determine whether planktonic synergistic interactions translate into early-stage antibiofilm efficacy. Thymol, eugenol, trans-cinnamaldehyde, and terpinen-4-ol were tested against *Streptococcus pneumoniae*, *Streptococcus pyogenes*, *Haemophilus influenzae*, *Haemophilus parainfluenzae*, *Moraxella catarrhalis*, methicillin-resistant *Staphylococcus aureus* (MRSA), and *Pseudomonas aeruginosa*. **Methods:** Minimum inhibitory concentrations were determined by broth microdilution. Synergistic interactions with clinically relevant antibiotics were assessed using the checkerboard method and fractional inhibitory concentration index (FICI) analysis. Selected combinations were further evaluated in a 6 h crystal violet-based early-stage biofilm model. Gram-positive strains generally exhibited higher susceptibility to the tested components than Gram-negative bacteria. **Results:** Synergistic interactions (FICI ≤ 0.5) were most frequently observed between β-lactam antibiotics and phenolic components, particularly thymol and trans-cinnamaldehyde. Strong synergy was detected for vancomycin-eugenol against MRSA and for amoxicillin/clavulanic acid–cinnamaldehyde against *M. catarrhalis*. Importantly, synergistic combinations translated into significantly enhanced inhibition of early biofilm formation, increasing inhibition rates by 15–40% compared to antibiotic monotherapy (*p* < 0.05). Selected essential oil components enhanced the antibacterial activity of clinically relevant antibiotics and effectively potentiated early-stage biofilm inhibition. **Conclusions:** These findings support further investigation of phytochemical-antibiotic combinations as potential adjunct strategies in respiratory infection management.

## 1. Introduction

Respiratory tract infections (RTIs), including both upper respiratory tract infections (URTIs) and lower respiratory tract infections (LRTIs), represent a significant global health burden, ranking among the leading reasons for outpatient visits and antimicrobial prescriptions [1,2]. While many RTIs are self-limiting due to their viral etiology, bacterial respiratory infections can lead to severe complications, prolonged morbidity, and substantial healthcare costs [3,4]. Of particular concern, the widespread use of antibiotics for RTIs exerts a selective pressure that fosters the development and dissemination of antimicrobial resistance, thereby compromising treatment efficacy and intensifying the need for antibiotic-sparing and combination strategies [5,6,7,8]. A broad spectrum of bacterial pathogens has been implicated in URTIs and respiratory complications. Key players include *Streptococcus pneumoniae*, *Haemophilus influenzae*, and *Moraxella catarrhalis*, which frequently colonize the upper airway and are major causes of acute otitis media and sinusitis [9]. Additionally, *Streptococcus pyogenes* is the primary bacterial pathogen responsible for pharyngitis and tonsillitis, while *Staphylococcus aureus* (including methicillin-resistant strains, MRSA) and *Pseudomonas aeruginosa* are crucial in complicated respiratory infections and persistent airway colonization [3,10,11]. These microorganisms have developed mechanisms to adhere to mucosal surfaces and medical devices, persist within the respiratory tract, and evade host immune responses, contributing to recurrent and chronic infections, especially in susceptible populations [12,13,14,15,16]. A key factor underlying persistence and therapeutic failure in these infections is the formation of bacterial biofilms. Biofilms are highly organized microbial communities embedded within a self-produced extracellular matrix, which confers substantial protection against both host immune defenses and antimicrobial agents [12,17,18]. Within this protected environment, bacteria adopt altered metabolic states and gene expression profiles, further enhancing their tolerance to antibiotics and promoting the development of chronic infections [19,20]. Notably, early-stage biofilms are clinically significant since the processes of bacterial adherence and microcolony formation are crucial for colonization and infection establishment. Interventions that can prevent or disrupt biofilm development at this critical point may offer considerable therapeutic advantages [21,22,23,24,25].

In this context, essential oils (EOs) and their major components have garnered significant interest as natural antimicrobial agents and potential antibiotic adjuvants in contemporary medical research [8,26,27]. Unlike conventional antibiotics that often target specific bacterial pathways, components of EOs generally exhibit multi-target effects, disrupting cellular membranes, enhancing permeability, interfering with bacterial stress responses, and modulating virulence factors, including biofilm formation [28,29]. Moreover, accumulating evidence indicates that combinations of EO components with antibiotics may enhance antibacterial efficacy, reduce the required antibiotic dose, and potentially expand antimicrobial spectra, thereby supporting their potential role in adjuvant-based strategies for the management of respiratory infections [8,30]. For example, a study on *Geophila repens* EO reported that its combinations with conventional antibiotics exerted broad-spectrum antibacterial effects, including activity against *P. aeruginosa* and *Escherichia coli*, with synergistic interactions frequently observed across multiple pathogen–antibiotic pairings [31]. From a component-focused perspective, thymol, eugenol, trans-cinnamaldehyde, and terpinen-4-ol are all small, lipophilic, plant-derived antimicrobials common in EOs, and their antibacterial action is largely membrane-centric [32,33]. They share hydrophobicity that promotes rapid partitioning into bacterial lipid bilayers, with functional groups (phenolic in thymol and eugenol; aldehyde in trans-cinnamaldehyde; alcohol in terpinen-4-ol) that enable membrane perturbation, disruption of membrane integrity, and perturbation of energy metabolism [32,34]. This membrane targeting is frequently accompanied by intracellular stress and increased permeability, facilitating synergistic interactions with other EO components or antibiotics [32,33]. Despite these encouraging findings, limited information is available regarding whether the synergistic antibacterial effects of EO components with antibiotics can be consistently translated into clinically relevant respiratory pathogens. Accordingly, the primary aim of this study was to evaluate the antibiotic adjuvant potential of thymol, eugenol, trans-cinnamaldehyde, and terpinen-4-ol against respiratory pathogens in both planktonic and early-stage biofilm forms. The four investigated components were selected based on the previously demonstrated strong antimicrobial and antibiofilm activities of EOs in which these compounds are the major constituents, including thyme, clove, Ceylon cinnamon bark, and tea tree oils [8,26,30,35,36]. 

## 2. Results

### 2.1. Antibiotic Susceptibility Profile

Disk diffusion-based antibiotic susceptibility testing was performed to determine the sensitivity of the respiratory bacteria included in this study to various antibiotics (Table 1). *H. influenzae* and *H. parainfluenzae* exhibited resistance to ampicillin while remaining susceptible to ceftriaxone and ciprofloxacin. This resistance pattern is consistent with the production of β-lactamase enzymes that hydrolyze ampicillin, whereas susceptibility to clavulanate indicates inhibition of β-lactamase activity [37,38]. Similarly, *M. catarrhalis* showed resistance to ampicillin but remained susceptible to amoxicillin/clavulanic acid, which is in agreement with its well-known β-lactamase production [39]. The MRSA strain demonstrated the expected resistance profile, characterized by resistance to oxacillin and penicillin, while remaining susceptible to vancomycin. This observation is consistent with the *mecA*-mediated resistance mechanism, which results in penicillin-binding proteins with reduced affinity for β-lactam antibiotics while preserving susceptibility to non-β-lactam agents [40,41]. *P. aeruginosa* was susceptible to antipseudomonal agents, including ceftazidime and piperacillin/tazobactam, confirming the clinical relevance of these antibiotics in the treatment of infections caused by this intrinsically resistant pathogen [42,43]. Both *S. pneumoniae* and *S. pyogenes* were susceptible to penicillin, consistent with their typical susceptibility profiles in clinical settings [37].

### 2.2. Minimum Inhibitory Concentrations

To quantitatively evaluate the antimicrobial activity of the tested antibiotics and EO components, minimum inhibitory concentration (MIC) determinations were performed against the investigated respiratory pathogens (Table 2). Among the tested strains, *S. pyogenes* showed the highest overall susceptibility, whereas *P. aeruginosa* was the most resistant species. Gram-positive bacteria demonstrated greater sensitivity compared to Gram-negative strains, which may be attributed to the presence of an outer membrane in Gram-negative bacteria acting as an additional permeability barrier [44,45].

Terpinen-4-ol showed the strongest antibacterial activity against streptococci, with particularly low MIC values against *S. pyogenes* (e.g., 19 µg/mL) and *S. pneumoniae*. Thymol demonstrated pronounced activity against MRSA (e.g., 156 µg/mL) and *M. catarrhalis*, while trans-cinnamaldehyde exhibited relatively enhanced activity against *Haemophilus* spp. In contrast, eugenol generally showed comparatively weaker antibacterial activity across most tested strains, while trans-cinnamaldehyde demonstrated variable but notably strong activity against certain respiratory pathogens, particularly *Haemophilus* spp.

### 2.3. Synergistic Interactions Between Antibiotics and Essential Oil Components

Checkerboard microdilution assays were performed to evaluate synergistic interactions between antibiotics and EO components against respiratory bacterial pathogens (Table 3). Synergy (FICI ≤ 0.5) was observed for multiple combinations, while antagonism was not detected. In *H. influenzae* and *H. parainfluenzae*, cinnamaldehyde–ceftriaxone combinations consistently showed synergy (FICI = 0.50), with additional synergy noted for thymol–ceftriaxone in *H. parainfluenzae* (FICI = 0.37). MRSA displayed pronounced synergy with eugenol–vancomycin (FICI = 0.18) and thymol–vancomycin (FICI = 0.25). In *M. catarrhalis*, cinnamaldehyde–amoxicillin/clavulanic acid (FICI = 0.18) and thymol–amoxicillin/clavulanic acid (FICI = 0.31) were synergistic.

In the case of *P. aeruginosa*, synergy was limited to thymol–amikacin and terpinen-4-ol–amikacin (both FICI = 0.37), whereas most combinations were additive. In Gram-positive *S. pneumoniae* and *S. pyogenes*, thymol and terpinen-4-ol combinations with β-lactam antibiotics consistently exhibited synergy (FICI 0.31–0.37).

### 2.4. Antibiofilm Assay

To assess the effects of the selected antibiotic-EO component combinations beyond planktonic cultures, we performed biofilm inhibition assays. Essential oil components alone exhibited moderate to strong antibiofilm activity, with inhibition ranging from approximately 65% to 85%. In contrast, antibiotic monotherapy showed variable and, in some cases, limited efficacy. For example, ceftriaxone inhibited *S. pneumoniae* biofilm formation by ~39%, while amoxicillin–clavulanic acid reduced *M. catarrhalis* biofilm biomass by ~50%.

**Figure 1:**
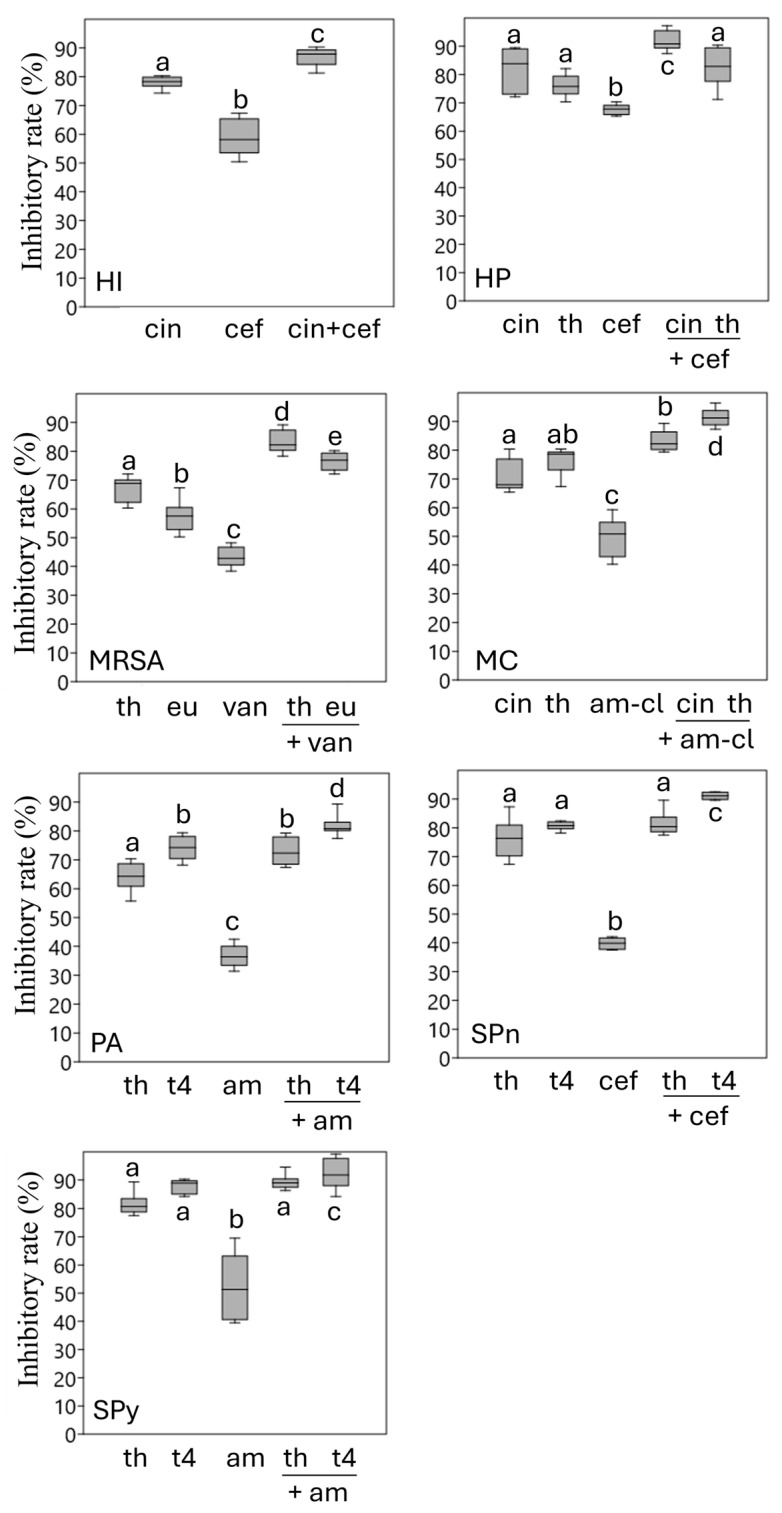
Early-stage (6 h) biofilm inhibition by antibiotic-essential oil component combinations against respiratory bacterial pathogens. Data were expressed using box plots with minimum to maximum values presented by vertical lines, with the median in the plot as a horizontal line. Different lowercase letters above or below the bars indicate significant differences between the means of the biofilm inhibition effect of the components according to Tukey’s test (*p* < 0.05), based on results of 8 parallel measurements (*n* = 8). Abbreviations: am (amoxicillin); am-cl (amoxicillin/clavulanic acid); cef (ceftriaxone); cin (cinnamaldehyde); eu (eugenol); t4 (terpinen-4-ol); th (thymol); van (vancomycin); HI (*H. influenzae)*; HP (*H. parainfluenzae*); MRSA (methicillin-resistant *S. aureus*); MC (*M. catarrhalis*); PA (*P. aeruginosa);* SPn (*S. pneumoniae*); SPy (*S. pyogenes*).

Notably, combinations identified as synergistic in planktonic checkerboard assays produced significantly greater biofilm inhibition than antibiotic monotherapy (*p* < 0.05). In *H. influenzae* and *H. parainfluenzae*, cinnamaldehyde combined with ceftriaxone increased biofilm inhibition to over 85–90%. In *M. catarrhalis*, the cinnamaldehyde–amoxicillin/clavulanic acid combination enhanced inhibition from ~50% to >80%. Among Gram-positive pathogens, potentiation was particularly pronounced: in *S. pneumoniae*, ceftriaxone combined with thymol or terpinen-4-ol increased inhibition from ~39% to >85–90%, and in *S. pyogenes*, thymol- or terpinen-4-ol-based combinations improved amoxicillin-mediated inhibition from ~52% to nearly 90%. Among the tested combinations, thymol combined with amoxicillin–clavulanic acid exhibited the highest antibiofilm activity against *M. catarrhalis*, reaching an inhibition rate of 91.42% (Figure 1).

## 3. Discussion

The increasing incidence of antimicrobial resistance in respiratory tract infections necessitates the development of alternative therapeutic strategies capable of enhancing antibiotic efficacy while potentially reducing the required doses. In this context, selected EO components, namely thymol, eugenol, trans-cinnamaldehyde, and terpinen-4-ol, were chosen based on the previously demonstrated strong antibacterial and antibiofilm activities of their corresponding EOs and represent promising adjuvant candidates due to their multi-target mechanisms of action and their relatively low propensity to induce resistance [36,46]. The present study systematically evaluated the antibiotic-adjuvant potential of four major EO components against clinically relevant respiratory bacterial pathogens by integrating planktonic susceptibility testing, checkerboard synergy analysis, and early-stage biofilm validation. The checkerboard microdilution assays demonstrated notable synergistic interactions between EO components and conventional antibiotics against several respiratory pathogens. A consistent synergistic effect was observed for the cinnamaldehyde–ceftriaxone combination (FICI = 0.50) in *Haemophilus* species. This interaction may be explained by the ability of cinnamaldehyde to disrupt bacterial cell envelopes, thereby increasing membrane permeability and facilitating ceftriaxone penetration, which may help overcome bacterial resistance mechanisms [47]. Notably, pronounced synergistic interactions were also observed in MRSA when combined with eugenol (FICI = 0.18) and thymol (FICI = 0.25). These findings corroborate previous reports demonstrating enhanced antibacterial activity of EO components against MRSA [48,49]. Such combined strategies may exploit not only the intrinsic antibacterial properties of these components but also their ability to modulate bacterial resistance-related processes, including mechanisms such as efflux activity, thereby enhancing the efficacy of conventional antibiotics [50]. In the case of *M. catarrhalis*, the synergistic interactions observed for cinnamaldehyde combined with amoxicillin/clavulanic acid (FICI = 0.18) and thymol combined with amoxicillin–clavulanic acid (FICI = 0.31) are particularly noteworthy. These results are consistent with the well-documented resistance patterns of *M. catarrhalis*, where β-lactamase production frequently limits the effectiveness of standard therapies. The use of EO components in combination with β-lactams may contribute to the modulation of resistance-related mechanisms [51,52]. In contrast, only limited synergistic effects were observed for *P. aeruginosa*, primarily restricted to the thymol–amikacin (FICI = 0.37) and terpinen-4-ol–amikacin combinations. These observations likely reflect the multifactorial intrinsic resistance mechanisms of *P. aeruginosa*, including reduced outer membrane permeability and active efflux systems, which may limit the efficacy of EO-based adjuvant strategies. Previous studies have demonstrated that the outer membrane of *P. aeruginosa* acts as a significant permeability barrier to numerous antimicrobial agents, including EO components and antibiotics [53,54]. In Gram-positive pathogens, such as *S. pneumoniae* and *S. pyogenes*, consistent synergistic effects were observed for combinations of thymol and terpinen-4-ol with β-lactam antibiotics (FICI range: 0.31–0.37). These findings further support the potential of EO components as adjunctive agents in the treatment of infections where β-lactam resistance is of increasing concern. The ability of these components to enhance the bactericidal activity of β-lactams provides a compelling basis for future investigations aimed at elucidating their combined therapeutic potential and underlying mechanisms of action.

In this context, it should be noted that, although a broader panel of antibiotics was included in the initial susceptibility screening, only selected antibiotics were further evaluated in MIC and checkerboard assays. This targeted selection was based on their clinical relevance for the investigated pathogens, as well as their therapeutic importance in respiratory infection management, allowing for a focused assessment of synergistic interactions with EO components. For example, vancomycin was prioritized for MRSA due to its established role as a clinically relevant therapeutic option, while β-lactam antibiotics and amikacin were selected for other strains based on their relevance in current treatment practice.

The results of the biofilm inhibition assays further highlight the potential of combining EO components with antibiotics to combat biofilm-forming respiratory pathogens. Essential oil components alone demonstrated considerable antibiofilm activity, achieving inhibition rates between 65% and 85%. In contrast, antibiotic monotherapy showed variable and often limited efficacy. For example, ceftriaxone inhibited *S. pneumoniae* biofilm formation by only 39%, while amoxicillin–clavulanic acid reduced *M. catarrhalis* biofilm biomass by approximately 50%. These observations are consistent with previous reports emphasizing the limited effectiveness of conventional antibiotics against biofilm-associated infections [55,56]. In contrast, synergistic combinations resulted in significantly greater biofilm inhibition (*p* < 0.05). Notably, the cinnamaldehyde–ceftriaxone combination achieved high inhibition rates (>85–90%) against *H. influenzae* and *H. parainfluenzae*, supporting earlier findings that natural components can disrupt biofilm structure and enhance antibiotic penetration [57,58]. A comparable enhancement was observed for *M. catarrhalis*, where the addition of cinnamaldehyde increased the activity of amoxicillin/clavulanic acid from approximately 50% to over 80%, suggesting a potential restoration of β-lactam efficacy in β-lactamase-producing strains [59,60]. Similarly, in Gram-positive pathogens, marked potentiation was detected: combinations of ceftriaxone with thymol or terpinen-4-ol resulted in substantial biofilm inhibition (>85–90%) in *S. pneumoniae*, while in *S. pyogenes* these combinations increased inhibition from roughly 52% to nearly 90%. Collectively, these data support the concept that selected EO components may function as biofilm-targeting adjuvants, improving antibiotic performance under biofilm-associated growth conditions [61,62]. Among the evaluated combinations against MRSA, the thymol–vancomycin pairing yielded the most pronounced potentiation effect, reflected by markedly reduced MIC values and consistent synergistic interaction patterns.

Despite the promising in vitro findings, translation into clinical application requires careful evaluation. Comprehensive toxicological profiling, pharmacokinetic characterization, and validation in relevant in vivo infection models are necessary to determine safety, therapeutic window, and feasibility. Future studies should also address formulation strategies to optimize the stability, bioavailability, and controlled delivery of EO components when used as antibiotic adjuvants.

## 4. Materials and Methods

### 4.1. Bacterial Strains and Culture Conditions

The following clinically relevant respiratory pathogens were included in this study (Table 4): *Haemophilus influenzae* DSM 4690, *Haemophilus parainfluenzae* DSM 8978, *Moraxella catarrhalis* DSM 9143, methicillin-resistant *Staphylococcus aureus* (MRSA) ATCC 700698, *Pseudomonas aeruginosa* ATCC 27853, *Streptococcus pneumoniae* ATCC 20566, and *Streptococcus pyogenes* DSM 20565. All strains were stored at −80 °C in appropriate cryopreservation medium and subcultured twice prior to experiments. Bacterial strains were cultivated in Brain Heart Infusion (BHI) broth (Sigma-Aldrich, Darmstadt, Germany) as the primary liquid growth medium. Cultures were incubated at 37 °C for 12 h in a shaking incubator (C25 Incubator Shaker, New Brunswick Scientific, Edison, NJ, USA) at 60 rpm to obtain actively growing bacterial suspensions. For *H. influenzae* (DSM 4690) and *H. parainfluenzae* (DSM 8978), BHI broth was supplemented with 1% (*v*/*v*) Supplement B (Diagon Kft., Budapest, Hungary) and 15 µg/mL nicotinamide adenine dinucleotide (NAD) (NAD; Sigma-Aldrich, Darmstadt, Germany) to ensure optimal growth conditions. These cultures were incubated under identical temperature and shaking conditions.

### 4.2. Determination of Antibiotic Susceptibility Profile

The antibiotic susceptibility of the investigated respiratory pathogens was determined using the Kirby–Bauer disk diffusion method in accordance with Clinical and Laboratory Standards Institute (CLSI) guidelines (M02, [63]). Bacterial suspensions were adjusted to the turbidity of a 0.5 McFarland standard and uniformly spread onto agar plates. Mueller–Hinton agar (Oxoid Ltd., Hampshire, UK) was used for all strains except *H. influenzae* and *H. parainfluenzae*, for which chocolate agar supplemented with appropriate growth factors was applied. After drying, antibiotic disks (Oxoid Ltd., Hampshire, UK) were placed onto the agar surface. The following antibiotic disks were tested: amikacin (30 µg), ampicillin (10 µg), amoxicillin (10 µg), amoxicillin–clavulanic acid (20/10 µg), ceftazidime (10 µg), ceftriaxone (30 µg), ciprofloxacin (5 µg), erythromycin (15 µg), gentamicin (30 µg), imipenem (10 µg), levofloxacin (5 µg), oxacillin (1 µg), penicillin (1 µg), piperacillin–tazobactam (100/10 µg), and vancomycin (5 µg). Plates were incubated at 35–37 °C for 16–18 h. Inhibition zone diameters were measured in millimeters and interpreted according to CLSI breakpoint criteria. All tests were performed in triplicate.

### 4.3. Antibiotics and Essential Oil Components

The antibiotics evaluated in this study were selected based on their clinical relevance in respiratory tract infections. Amikacin (Likacin 250 mg/mL solution for injection; Lisapharma S.p.A, Erba, Italy), vancomycin (500 mg powder for solution for infusion; TEVA, Debrecen, Hungary), amoxicillin (powder for solution; specify manufacturer if applicable), amoxicillin–clavulanic acid (Amoxicillin/Clavulanic Acid Sandoz 1000 mg/200 mg powder for solution for injection; Sandoz GmbH, Kundl, Austria), and ceftriaxone (CEFTRIAXONE KABI 2 g powder for solution for infusion; Fresenius Kabi Deutschland GmbH, Bad Homburg, Germany) were used. Antibiotic stock solutions were prepared in sterile distilled water according to the declared active substance content and further diluted in the appropriate test medium to obtain the required concentrations for susceptibility testing. The EO components investigated included thymol (≥99% purity), eugenol (≥99%), trans-cinnamaldehyde (≥98%), and terpinen-4-ol (≥97%), all purchased from Sigma-Aldrich Ltd. (Darmstadt, Germany). Stock solutions were prepared using Tween 40 as a solubilizing agent. Following dilution in the microdilution assay, the final concentration of Tween 40 did not exceed 0.5% (*v*/*v*). Control wells containing 0.5% Tween 40 without active components were included in all experiments to confirm the absence of intrinsic antimicrobial activity.

### 4.4. Determination of Minimum Inhibitory Concentration (MIC)

Minimum inhibitory concentrations (MICs) of antibiotics and EO components were determined using the broth microdilution method in accordance with CLSI guidelines (CLSI M02; [63]). Based on the initial susceptibility profiles, one clinically relevant antibiotic per bacterial strain was selected for subsequent MIC and checkerboard assays. The selection was guided by their therapeutic relevance in respiratory tract infections and their established clinical use against the respective pathogens. Bacterial suspensions were prepared from fresh overnight cultures and adjusted spectrophotometrically to a 0.5 McFarland standard. The inoculum was subsequently diluted in the appropriate test medium to obtain a final bacterial concentration of approximately 1 × 10^5^ CFU/mL in each well of a sterile 96-well microtiter plate. Two-fold serial dilutions of antibiotics and components were prepared in BHI broth, with strain-specific supplements applied as described above. Each well contained 100 µL of the test compound dilution and 100 µL of bacterial suspension, resulting in a total volume of 200 µL. Tween 40 was used as a solubilizing agent. After dilution in the assay system, the final concentration of Tween 40 did not exceed 0.5% (*v*/*v*). Control wells containing medium only, inoculum only, and 0.5% Tween 40 without active components were included in all experiments. Microplates were incubated at 37 °C for 24 h under strain-specific atmospheric conditions. The MIC was defined as the lowest concentration of the tested component that showed no visible bacterial growth. All experiments were performed in triplicate and repeated at least three times independently.

### 4.5. Checkerboard Titration

Synergistic interactions between antibiotics and EO components were evaluated using the checkerboard broth microdilution method in sterile 96-well microtiter plates. Two-fold serial dilutions of each antibiotic were prepared along one axis, and two-fold serial dilutions of each EO component (thymol, eugenol, trans-cinnamaldehyde, and terpinen-4-ol) were prepared along the other axis. For both agents, the tested concentration range corresponded to 8 × MIC, 4 × MIC, 2 × MIC, 1 × MIC, 1/2 × MIC, 1/4 × MIC, and 1/8 × MIC, based on the MIC values determined for each strain. Each well contained 100 µL of the antibiotic–component mixture and 100 µL of the standardized bacterial inoculum, resulting in a final volume of 200 µL per well and a final inoculum of approximately 10^5^ CFU/mL. Plates were incubated at 37 °C for 24 h under strain-specific atmospheric conditions. Growth controls (medium + inoculum) and solvent controls (Tween 40 at a final concentration of 0.5% *v*/*v*) were included on each plate. Bacterial growth was quantified by measuring optical density at 600 nm (OD600) using a microplate reader. Growth inhibition was calculated relative to the untreated growth control [64] as

% inhibition = [1 − (OD_treated/OD_control)] × 100

For endpoint determination, the combination MIC was defined as the lowest antibiotic–component concentration pair resulting in ≥90% growth inhibition compared to the growth control. The fractional inhibitory concentration index (FICI) was calculated as

FICI = FIC_A + FIC_B,

where FIC_A = (MIC of the antibiotic in combination)/(MIC of the antibiotic alone), and FIC_B = (MIC of the component in combination)/(MIC of the component alone).

Interactions were interpreted as synergy (FICI ≤ 0.5), additive (0.5 < FICI ≤ 1.0), indifferent (1.0 < FICI ≤ 4.0), or antagonistic (FICI > 4.0) [65]. All checkerboard assays were performed in triplicate and repeated at least three times independently.

### 4.6. Early-Stage Biofilm Formation Assay

The effect of selected antibiotic–component combinations on early-stage biofilm formation was evaluated using a crystal violet microtiter plate assay. Fresh bacterial suspensions were prepared from overnight cultures and adjusted to the required inoculum density in the appropriate growth medium (1 × 10^8^ CFU/mL). Aliquots of 100 µL of bacterial suspension were added to sterile 96-well flat-bottom polystyrene microplates together with 100 µL of the test solutions, resulting in a final volume of 200 µL per well. The following conditions were tested: untreated control (growth control), antibiotic alone, component alone, and the selected antibiotic–component combinations identified as synergistic in the checkerboard assay. Solvent controls containing 0.5% Tween 40 were included in all experiments. Plates were incubated statically at 37 °C for 6 h under strain-specific atmospheric conditions to allow initial bacterial adhesion and early biofilm development. After incubation, non-adherent cells were gently removed, and wells were washed three times with sterile physiological saline. The attached biomass was fixed with methanol, followed by staining with 0.1% (*w*/*v*) crystal violet solution. Excess stain was removed by washing with distilled water, and the plates were air-dried. Bound crystal violet was solubilized with 33% (*v*/*v*) acetic acid, and absorbance was measured at 595 nm using a microplate reader. Antibiofilm activity is expressed as the inhibitory rate calculated according to the following equation:

Inhibitory rate (%) = (1 − S/C) × 100,

where C and S represent the mean absorbance values of the untreated control and treated sample groups, respectively [64]. All experiments were performed using eight parallel replicates.

### 4.7. Statistical Analysis

Statistical analyses were conducted using PAST software version 3.11 [66]. Differences in biofilm inhibition rates between treatment groups were evaluated by one-way analysis of variance (ANOVA). Upon detection of a significant overall effect, pairwise comparisons were performed using Tukey’s honestly significant difference (HSD) post hoc test. Statistical significance was set at *p* < 0.05.

## 5. Conclusions

The present study demonstrates that selected EO components enhance the antibacterial efficacy of clinically relevant antibiotics against respiratory pathogens. Importantly, planktonic synergistic interactions translated into improved inhibition of early-stage biofilm formation. These findings highlight the potential of EO components as antibiotic adjuvants and support further investigation of their translational applicability in respiratory infection management.

## Figures and Tables

**Table 1:** Disk diffusion-based antibiotic susceptibility profiles of clinically relevant respiratory bacterial pathogens.

Antibiotics	HI	HP	MRSA	MC	PA	SPn	SPy
Amikacin	S	S	-	-	S	-	-
Amoxicillin	-	-	R	-	-	-	S
Amoxicillin/clavulanic acid	-	-	R	S	S	S	S
Ampicillin	R	R	R	R	-	-	-
Ceftriaxone	S	S	R	-	-	-	-
Ceftazidime	-	-	R	-	S	-	-
Ciprofloxacin	S	S	R	R	S	S	S
Erythromycin	-	-	-	-	-	R	-
Gentamicin	-	-	S	-	S	-	-
Imipenem	S	S	-	S	S	S	S
Levofloxacin	-	-	-	-	-	-	-
Oxacillin	-	-	R	-	-	-	-
Penicillin	-	-	R	-	-	S	S
Piperacillin/tazobactam	-	-	-	-	S	-	-
Vancomycin	-	-	S	-	-	-	-

R (resistant); S (susceptible); “-” (not tested/not applicable due to limited clinical relevance for the given bacterial strain); HI (*H. influenzae*); HP (*H. parainfluenzae*); MRSA (methicillin-resistant *S. aureus*); MC (*M. catarrhalis*); PA (*P. aeruginosa*); SPn (*S. pneumoniae*); SPy (*S. pyogenes*).

**Table 2:** Minimum inhibitory concentrations (MICs) of antibiotics and essential oil components against clinically relevant respiratory bacterial pathogens.

	HI	HP	MRSA	MC	PA	SPn	SPy
amikacin	-	-	-	-	8	-	-
amoxicillin	-	-	-	-	-	-	0.006
amoxicillin–clavulanic acid	-	-	-	0.5	-	-	-
ceftriaxone	0.5	0.5	-	-	-	1	-
vancomycin	-	-	2	-	-	-	-
cinnamaldehyde	19	19	625	313	625	78	39
eugenol	78	78	313	156	625	156	78
thymol	39	19	156	78	156	78	19
terpinen-4-ol	78	39	313	313	313	39	19

MIC values are expressed in µg/mL. Amoxicillin/clavulanic acid was tested at a fixed 5:1 ratio (as in the clinical formulation). MIC values are reported as the total concentration of the combination. HI (*H. influenzae*); HP (*H. parainfluenzae*); MRSA (methicillin-resistant *S. aureus*); MC (*M. catarrhalis*); PA (*P. aeruginosa*); SPn (*S. pneumoniae*); SPy (*S. pyogenes*).

**Table 3:** Fractional inhibitory concentration index (FICI) values of antibiotic–essential oil component combinations against respiratory bacterial pathogens.

Bacterial Strain	Components	MIC Combination	FICI	Interaction
HI	cinnamaldehyde	4.8	0.50	**SYN**
	ceftriaxone	0.1		
	eugenol	19.5	0.75	ADD
	ceftriaxone	0.3		
	thymol	9.8	0.75	ADD
	ceftriaxone	0.3		
	terpinen-4-ol	19.5	0.75	ADD
	ceftriaxone	0.3		
HP	cinnamaldehyde	4.9	0.50	**SYN**
	ceftriaxone	0.2		
	eugenol	39	1.00	ADD
	ceftriaxone	0.3		
	thymol	2.4	0.37	**SYN**
	ceftriaxone	0.1		
	terpinen-4-ol	9.8	0.75	ADD
	ceftriaxone	0.3		
MRSA	cinnamaldehyde	156.2	0.75	ADD
	vancomycin	1.0		
	eugenol	19.5	0.18	**SYN**
	vancomycin	0.3		
	thymol	19.5	0.25	**SYN**
	vancomycin	0.5		
	terpinen-4-ol	0.1562	0.75	ADD
	vancomycin	0.5		
MC	cinnamaldehyde	19.5	0.18	**SYN**
	amoxicillin–clavulanic acid	0.0625		
	eugenol	39	0.75	ADD
	amoxicillin-clavulanic acid	0.25		
	thymol	4.8	0.31	**SYN**
	amoxicillin–clavulanic acid	0.125		
	terpinen-4-ol	78	0.75	ADD
	amoxicillin–clavulanic acid	0.25		
PA	cinnamaldehyde	156.2	0.75	ADD
	amikacin	4		
	eugenol	312.5	0.63	ADD
	amikacin	1		
	thymol	19	0.37	**SYN**
	amikacin	2		
	terpinen-4-ol	78	0.37	**SYN**
	amikacin	1		
SPn	cinnamaldehyde	39	1.00	ADD
	ceftriaxone	0.5		
	eugenol	19.5	0.63	ADD
	ceftriaxone	0.5		
	thymol	9.75	0.37	**SYN**
	ceftriaxone	0.25		
	terpinen-4-ol	4.8	0.37	**SYN**
	ceftriaxone	0.25		
SPy	cinnamaldehyde	19.5	1.00	ADD
	amoxicillin	0.003		
	eugenol	19.5	0.75	ADD
	amoxicillin	0.003		
	thymol	2.43	0.37	**SYN**
	amoxicillin	0.0015		
	terpinen-4-ol	1.21	0.31	**SYN**
	amoxicillin	0.0015		

SYN (synergistic effect); ADD (additive effect); HI (*H. influenzae*); HP (*H. parainfluenzae*); MRSA (methicillin-resistant *S. aureus*); MC (*M. catarrhalis*); PA (*P. aeruginosa*); SPn (*S. pneumoniae*); SPy (*S. pyogenes*). Combination MIC values are expressed in µg/mL. Bold values indicate synergistic interactions (FICI ≤ 0.5).

**Table 4:** Bacterial strains used in the study and their strain identifiers.

Bacterial Strain	Strain Identifier
*Haemophilus influenzae*	DSM 4690
*Haemophilus parainfluenzae*	DSM 8978
Methicillin-resistant *Staphylococcus aureus* (MRSA)	ATCC 700698
*Moraxella catarrhalis*	DSM 9143
*Pseudomonas aeruginosa*	ATCC 27853
*Streptococcus pneumoniae*	ATCC 20566
*Streptococcus pyogenes*	DSM 20565

## Data Availability

The data supporting the findings of this study are available from the corresponding author upon reasonable request.
